# In the national epidemiological bulletins – a selection from current issues

**DOI:** 10.2807/1560-7917.ES.2016.21.40.30365

**Published:** 2016-10-06

**Authors:** Eurosurveillance editorial team

**Affiliations:** 1 European Centre for Disease Prevention and Control (ECDC), Stockholm, Sweden

**Keywords:** public health

**Respiratory diseases**
Seasonal flu vaccine uptake in persons aged 65 years and older
Epi-Insight 2016;17(10)
October 2016, Ireland
http://ndsc.newsweaver.ie/epiinsight/pnpyh4q4puin0ykqgxo0gg?a=1&p=50883926&t=17517774
**Food- and waterborne diseases**
Sharp increase in campylobacter infection
Folkhälsomyndigheten website
3 October 2016, Sweden
https://www.folkhalsomyndigheten.se/nyheter-och-press/nyhetsarkiv/2016/oktober/kraftig-okning-av-infektion-med-campylobacter/
An oyster-associated norovirus outbreak following a corporate dinner – cohort study, Toulouse (France), January 2015
Bulletin épidémiologique hebdomadaire, 26–27
6 September 2016, France
http://invs.santepubliquefrance.fr/beh/2016/26-27/pdf/2016_26-27.pdf
**Healthcare-associated infections and antibiotic resistance**
Surveillance of *Candidaemia* in England, Wales and Northern Ireland: 2015
Health Protection Report; 10(32)
23 September 2016, UK
https://www.gov.uk/government/uploads/system/uploads/attachment_data/file/555332/hpr3216_cnddm.pdf
Voluntary surveillance of bacteraemia caused by *Pseudomonas* spp. and *Stenotrophomonas* spp. in England: 2008–2015Health Protection Report; 10(32)
23 September 2016, UK
https://www.gov.uk/government/uploads/system/uploads/attachment_data/file/555333/hpr3216_psdmns.pdf

**Vaccine-preventable diseases**
Immunisation – measles, mumps rubella, whooping cough and vaccine uptake
HPS Weekly Report, 2016;50(40)
4 October 2016, Scotland
http://www.hps.scot.nhs.uk/documents/ewr/pdf2016/1640.pdf
Quarterly vaccination coverage statistics for children aged up to five years in the UK (COVER programme): April to June 2016
Health Protection Report; 10(33)
30 September 2016, UK
https://www.gov.uk/government/uploads/system/uploads/attachment_data/file/556895/HPR_COVER_Q1617_1_April_to_June_2016_v4.pdf
Laboratory confirmed reports of invasive meningococcal disease in England: April to June 2016Health Protection Report; 10(33)
30 September 2016, UK
https://www.gov.uk/government/uploads/system/uploads/attachment_data/file/557329/hpr3316_IMD.pdf
Preliminary vaccine coverage estimates for the meningococcal B (MenB) immunisation programme for England, update to the end of August 2016
Health Protection Report; 10(32)
23 September 2016, UK
https://www.gov.uk/government/uploads/system/uploads/attachment_data/file/555057/hpr3216_menB.pdf
National rotavirus immunisation programme update: preliminary vaccine coverage for England, February 2016 to July 2016Health Protection Report; 10(32)
23 September 2016, UK
https://www.gov.uk/government/uploads/system/uploads/attachment_data/file/555048/hpr3216_rtvrs_VC.pdf
Vaccine coverage estimate for the GP based catch-up meningococcal ACWY (MenACWY) immunisation programme for school leavers (becoming 18 before 31 August 2016) in England, cumulative data to end-August 2016
Health Protection Report; 10(32)
19 September 2016, UK
https://www.gov.uk/government/uploads/system/uploads/attachment_data/file/553736/hpr3216_menACWY_VCa.pdf
Meningococcal disease in 2015
EPI-NEWS 37, 2016
14 September 2016, Denmark
http://www.ssi.dk/English/News/EPI-NEWS/2016/No%2037%20-%202016.aspx
Measles cases in the Copenhagen area
EPI-NEWS 36, 2016
7 September 2016, Denmark
http://www.ssi.dk/English/News/EPI-NEWS/2016/No%2036%20-%202016.aspx
**Sexually transmitted diseases**
Syphilis in Scotland 2015: update
HPS Weekly Report, 2016;50(39)
27 September 2016, Scotland
http://www.hps.scot.nhs.uk/documents/ewr/pdf2016/1639.pdf
*Chlamydia trachomatis* and *Neisseria gonorrhoeae* infection in Scotland: laboratory diagnoses 2006 – 2015
HPS Weekly Report, 2016;50(39)
27 September 2016, Scotland
http://www.hps.scot.nhs.uk/documents/ewr/pdf2016/1639.pdf
Gonococcal antibiotic surveillance in Scotland (GASS): prevalence, patterns and trends in 2015
HPS Weekly Report, 2016;50(39)
27 September 2016, Scotland
http://www.hps.scot.nhs.uk/documents/ewr/pdf2016/1639.pdf
Gonorrhoea 2015
EPI-NEWS 38, 2016
21 September 2016, Denmark
http://www.ssi.dk/English/News/EPI-NEWS/2016/No%2038%20-%202016.aspx
Syphilis 2015
EPI-NEWS 36, 2016
7 September 2016, Denmark
http://www.ssi.dk/English/News/EPI-NEWS/2016/No%2036%20-%202016.aspx
**Zoonoses and vector-borne diseases**
Incidence of protozoal infection reported to HPS in 2015
HPS Weekly Report, 2016;50(37)
13 September 2016, Scotland
http://www.hps.scot.nhs.uk/documents/ewr/pdf2016/1637.pdf
General bacterial and protozoal outbreaks of infectious intestinal disease reported to HPS in 2015
HPS Weekly Report, 2016;50(36)
6 September 2016, Scotland
http://www.hps.scot.nhs.uk/documents/ewr/pdf2016/1636.pdf
Surveillance of Shiga-toxin producing *Escherichia coli* (STEC) in the Netherlands, 2015
Infectieziekten Bulletin 2016; 27(7)
September 2016, the Netherlands
http://www.rivm.nl/dsresource?objectid=rivmp:321901&type=org&disposition=inline&ns_nc=1
**Hepatitides**
Hepatitis E outbreak associated with the consumption of a spit-roasted piglet, Brittany (France), 2013
Bulletin épidémiologique hebdomadaire, 26–27
6 September 2016, France
http://invs.santepubliquefrance.fr/beh/2016/26-27/pdf/2016_26-27.pdf
Hepatitis B vaccination for injecting drug users: from vaccination programme to individual care
Infectieziekten Bulletin 2016; 27(7)
September 2016, the Netherlands
http://www.rivm.nl/dsresource?objectid=rivmp:321901&type=org&disposition=inline&ns_nc=1
**Other**
Pseudomonas infections associated with ear-piercing after-care products
Health Protection Report; 10(31)
16 September 2016, UK
https://www.gov.uk/government/publications/health-protection-report-volume-10-2016/hpr-volume-10-issue-31-news-16-september

